# Water Quality Criteria and Ecological Risk Assessment of Fluoride for the Protection of Water Organisms in Surface Water

**DOI:** 10.3390/toxics14010106

**Published:** 2026-01-22

**Authors:** Jiahao Zhang, Yuting Pu, Jing Ye, Xiaojun Hu, Chenglian Feng

**Affiliations:** 1 Faculty of Chemical Engineering and Energy Technology, Shanghai Institute of Technology, Shanghai 201418, China; 246062220@mail.sit.edu.cn (J.Z.); yejinganna@sit.edu.cn (J.Y.); hu-xj@sit.edu.cn (X.H.); 2 State Key Laboratory of Environmental Criteria and Risk Assessment, Chinese Research Academy of Environmental Sciences, Beijing 100012, China; yuting_pu@163.com; 3 College of Water Sciences, Beijing Normal University, Beijing 100875, China

**Keywords:** fluoride, water quality criteria, risk assessment, species sensitivity method, risk quotient

## Abstract

The widespread occurrence of fluoride pollution in water bodies and its toxic effects on aquatic organisms have raised significant environmental concerns; however, studies on water quality criteria for fluoride remain relatively limited. This study aimed to derive such criteria and assess the ecological risks of fluoride in China’s surface waters, for the reference of readers. Acute and chronic toxicity data were collected globally, covering 34 species (14 families, 4 phyla) and 7 species (5 families, 3 phyla), respectively. Using species sensitivity distribution (SSD) methods, the short-term water quality criterion (SWQC) and long-term water quality criterion (LWQC) were derived as 17.47 mg/L and 3.334 mg/L. Ecological risk assessment based on the risk quotient (RQ) identified several high-risk areas among 32 major river and lake basins, with RQ values of 6.326 (Xihe River), 1.953 (Ebinur Lake), 1.368 (Chagan Lake), and 1.158 (Shahe River). At the provincial level, Guangxi Zhuang Autonomous Region showed as no risk (RQ = 0.0001140), while other regions were classified as moderate or low risk. This study achieved its objectives of deriving water quality criteria for fluoride and conducting an ecological risk assessment for surface waters in China. It also highlights current limitations, including insufficient fluoride toxicity data and the frequent oversight of key indicators in existing assessments. Future research could focus on improving water quality criteria derivation and risk assessment methods through integrated predictive modeling and expanded toxicity datasets.

## 1. Introduction

Fluorine is a common element in nature, first discovered by German scholar Georgius Agricola in the form of fluorite (calcium fluoride, CaF_2_) in 1529 [1]. Broadly speaking, fluoride refers to compounds containing fluorine elements, but in academia, fluoride usually refers to ionic fluorides (mostly inorganic fluorides). Fluoride originates from volcanic eruptions, rock weathering, and industrial emissions, existing as particulate matter (e.g., Al_2_(SiF_6_)_3_, CaF_2_) in soil and water, and as gaseous forms (e.g., HF, SiF_4_) in the atmosphere [2,3]. Industrial sources such as coal-fired plants and metallurgical factories emit HF and fluoride-containing particulates, with HF being a major emission due to its widespread use in chemical, material, and energy industries [4]. Fluorine has a wide range of harmful effects on living organisms, causing damage to various organ systems in the human body, leading to diseases, e.g., vascular inflammation and hardening, tooth and bone mineralization. Animals consuming plants or feed with high fluoride content may experience similar symptoms; plants will experience slow development of their roots, leaves, buds, and other tissues and organs after being enriched with fluoride, resulting in damage during branching, fruiting, and other growth stages [5,6,7,8]. The nature of fluorosis varies in different countries and regions in the world. It is mainly divided into drinking water type fluorosis, tea drinking type fluorosis, and coal burning pollution type fluorosis. Drinking water type fluorosis is widespread in all countries in the world. Tea drinking type fluorosis is mainly distributed in China, Spain, Vietnam, Britain, and Jordan, and coal burning pollution type fluorosis is unique to China, mainly in the North China Plain and provincial-level administrative regions in the south [9,10,11,12,13,14,15,16,17,18,19]. In addition, various endemic fluorosis caused by inorganic fluorides have not been effectively controlled and managed. In China, for example, dental fluorosis was detected in 8.61% of children across 28 provincial-level regions as of 2023, while skeletal fluorosis was reported in 8.52% of adults in seven regions [20]. Similarly, data from the 2004 U.S. National Health and Nutrition Examination Survey (NHANES) showed a dental fluorosis prevalence of 23% nationwide, with the rate among adolescents reaching 41% [21]. These figures demonstrate that fluorosis remains a persistent public health concern across different populations.

The toxic effects of fluoride on aquatic organisms are a key issue in studying the water pollution it causes. According to reports, fluoride can cause multiple types of damage to fish, including skin and other organs [22]. In an environment with an initial fluoride concentration of 7.2 mg/L and exposure for up to 120 h, the mortality rate of mussels can reach 30% [23]. It can be seen that it is necessary to establish scientific water quality criteria (WQC) to ensure appropriate protection of organisms. The water quality criteria refer to the maximum concentration or level of pollutants or harmful factors in the water environment that do not have harmful effects on human health or aquatic ecosystems [24]. At present, the water quality limits for ionic fluorides exist in the current standards of many countries. In China, the fluoride (calculated as F^−^) limit range specified in the “Surface Water Environment Quality Standard” (GB 3838-2002 [25]), “Groundwater Quality Standard” (GB/T 14848-2017 [26]), and “Standard for Drinking Water Quality” (GB 5749-2022 [27]) is 1.0 mg/L–2.0 mg/L. This level is largely consistent with fluoride limit values adopted in many countries, which may indicate that current standards are not always scientifically tailored to local biological specificity or actual usage needs. Furthermore, research on the toxic effects of fluoride on aquatic organisms and the corresponding water quality standards remains inadequate, leading to a significant lack of data regarding fluoride’s ecotoxicological impacts. This gap contrasts sharply with the observable toxic effects reported in current studies. Based on this, this study collected fluoride toxicity data in recent years and used the species sensitivity distribution (SSD) method to derive acute and chronic criteria. China was selected as the study area, and the risk quotient (RQ) method was used to assess the ecological risk of surface water within its territory, providing reference for research on fluoride limit values. Finally, we addressed the scientific uncertainties in data processing and recommended expanding the current dataset on the aquatic toxicity of fluorides. To enhance the objectivity of fluoride criteria, we further proposed the incorporation of appropriate modifying factors during data analysis.

## 2. Materials and Methods

### 2.1. Collection and Screening of Toxicity Data

The principles of data collection and screening follow the Chinese “Technical guideline for deriving water quality criteria for freshwater organisms” (HJ 831-2022) (hereinafter referred to as the “Guideline”), sourced from the ECOTOX database, Web of Science, China National Knowledge Infrastructure and other databases [28]. The collected data will be screened, and the specific screening content is as follows: (1) Due to the fact that the species involved in the collected data are not limited to native Chinese species, only some Chinese freshwater invasive species will be removed based on the relevant list in Appendix C of the guideline. (2) For the most suitable exposure time in experimental conditions, it is stipulated that for animal acute experiments, it is about 24 h for rotifers, 48 h for fleas and chironomatodes, and 96 h for other species. For plant acute experiments, it is about 96 h. For animal chronic experiments, it is not less than 48 h for rotifers, not less than 21 days for other animals, or covering a sensitive life cycle. For plant chronic experiments, it is not less than 21 days or covering one generation. (3) For the screening of exposure types, it is stipulated that static, semi-static, and flow-through can be selected for acute experiments, semi-static and flow-through can be selected for chronic experiments, and static and semi-static can be selected for microalgae. (4) Priority of toxicity data: 50% lethal concentration (LC_50_) and 50% effect concentration (EC_50_) can be used as acute toxicity endpoints without distinguishing priority. Chronic toxicity endpoints are maximum acceptable toxicant concentration (MATC) = chronic value for the same effect (CVE) > 20% effect concentration (EC_20_) > 10% effect concentration (EC_10_) = no observed effect concentration (NOEC) > lowest observed effect concentration (LOEC) > EC_50_ > LC_50_. Exposure endpoints are classified as flow-through exposure data > semi-static exposure data > static exposure data [29].

### 2.2. Species Sensitivity Distribution Method

**Figure 1:**
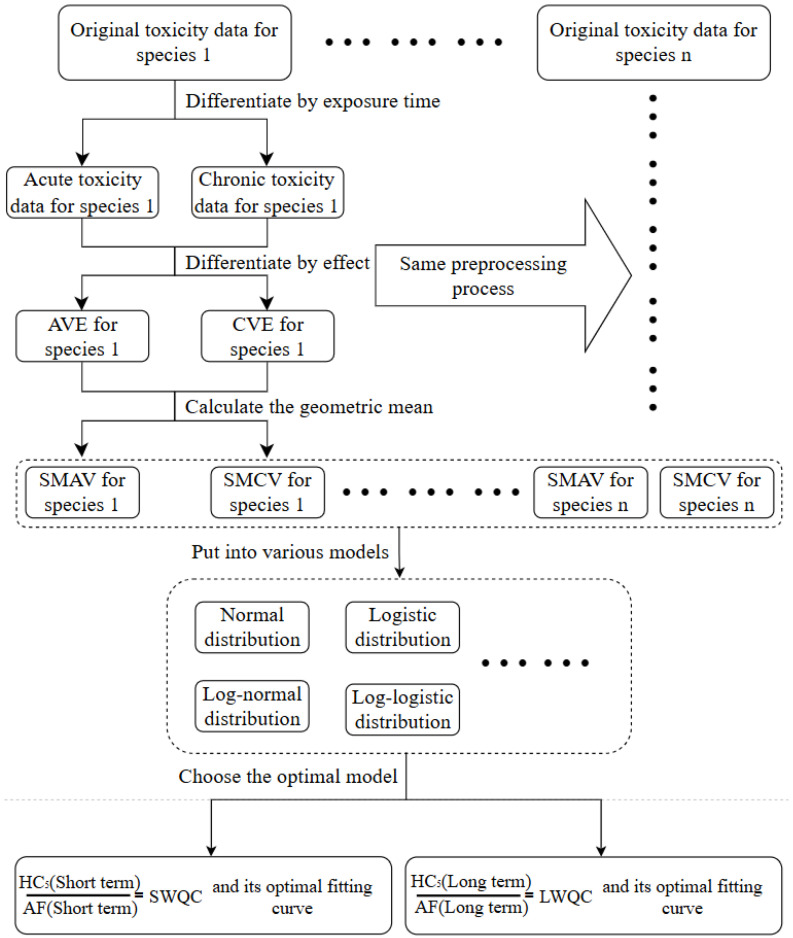
The operational process of species sensitivity distribution.

The species sensitivity distribution method is a recommended method for deriving water quality criteria of freshwater organisms in the guideline. It is a method that uses different models to fit the distribution of species sensitivity and calculate the pollutant concentration that can protect (100 − x)% of organisms, namely HCx (hazardous concentration for x% of species), and then extrapolates the assessment factor (AF) to obtain the criteria (i.e., the predicted no-effect concentration, PNEC) [30]. This method reflects the differences in sensitivity of different species to the same pollutant due to postnatal factors, e.g., lifestyle habits and geographical location in toxicology, and can use different optimal probability models to describe these sensitivities, making it easier to compare differences [31]. This method involves a series of data processing and selecting appropriate models for fitting, ultimately deriving short-term water quality criteria for aquatic organisms (SWQC) and long-term water quality criteria for aquatic organisms (LWQC). The specific steps are shown in Figure 1. The model with root mean square error (RMSE) closest to 0 (indicating the highest model fitting accuracy) and a probability *p*-value > 0.05 is considered as the optimal model. In SSD modeling, PNEC is conventionally derived using the hazardous concentration for 5% of species (HC_5_); it is also the most frequently applied hazard concentration in a wide range of environmental risk assessments and criteria studies [32,33]. The assessment factor (AF) accounts for uncertainties that may influence the final evaluation of toxicity. Its primary purpose is to quantify potential differences in toxic effects across varying environments or species, thereby supporting the extrapolation of reliable data to untested scenarios for predictive risk assessment [34]. According to the relevant standards stipulated by China, the selection of AF is based on the value of 3 when the number of species covered by toxicity data is less than or equal to 15, and 2 when the number is greater than 15 [35]. The fitting software used in this study is the Chinese “National Ecological Environment Criteria Calculation Software-Species Sensitivity Distribution Method” (EEC-SSD) (version 1.0).

### 2.3. Ecological Risk Assessment

The risk quotient method is a relatively simple ecological risk assessment method, mainly used for risk assessment of surface water. The calculation formula for this method is as follows:

(1) $$\rho_{\mathrm{WQC}}\;=\;\frac{{\mathrm{HC}}_{5}}{\mathrm{AF}},$$

(2) $$\mathrm{RQ}=\frac{\rho_{\mathrm{MEC}}}{\rho_{\mathrm{WQC}}}$$

In the formula, HC_5_ is the 5% species hazard concentration, measured in mg/L; AF is assessment factor, dimensionless; ρWQC is the derived water quality criteria concentration, measured in mg/L; ρMEC is the measured environmental concentration, and in this article, the collected concentration data is in mg/L; RQ is the risk quotient value, dimensionless. The basis for risk assessment is that RQ ≥ 1 indicates a high ecological risk of fluoride in the local water body, 1 > RQ ≥ 0.1 indicates a moderate ecological risk of fluoride in the local water body, 0.1 > RQ ≥ 0.01 indicates a low ecological risk of fluoride in the local water body, and RQ < 0.01 indicates no ecological risk of fluoride in the local water body [36].

## 3. Results

### 3.1. Collection and Screening of Toxicity Data

**Figure 2:**
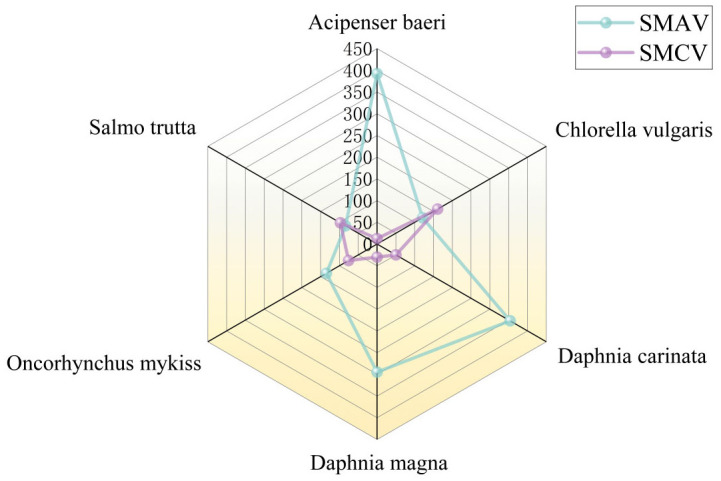
Comparison of SMAV and SMCV data for selected species.

The acute and chronic toxicity data compiled for fluoride were limited to three ionic forms: sodium fluoride, aluminum fluoride, and ammonium fluoride. The acute dataset covered 34 species across 14 families and 4 phyla, while the chronic dataset included 7 species from 5 families and 3 phyla. The geometric mean of the species mean acute value (SMAV) was 226.5 mg/L. The most sensitive species was *Hydropsyche bronta* (SMAV = 20.43 mg/L), and the least sensitive was *Lepomis macrochirus* (*Bluegill*, SMAV = 663.3 mg/L). For chronic toxicity, the geometric mean of the species mean chronic value (SMCV) was 63.60 mg/L. The most sensitive chronic species was *Acipenser baeri* (*Siberian sturgeon*, SMCV = 12.37 mg/L), and the least sensitive was *Chlorella vulgaris* (*Chlorella*, SMCV = 160.8 mg/L). Detailed results are provided in Table 1 and Table 2. Figure 2 presents a comparison of species for which both acute and chronic toxicity values are available.

### 3.2. Fluoride Criteria Derived from Species Sensitivity Distribution Method

**Figure 3:**
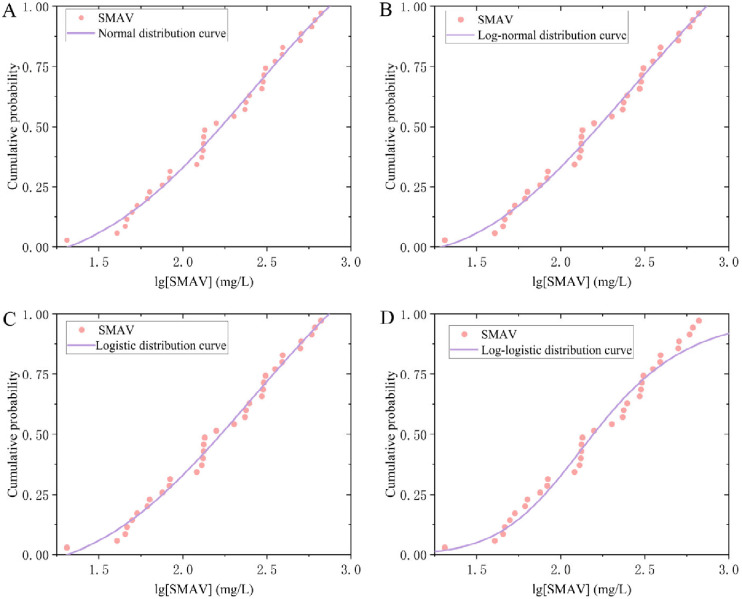
Fitting curves of four models to lg (SMAV) data. (**A**–**D**) represent the fitting curves using the normal distribution model, logarithmic normal distribution model, logistic distribution model, and logarithmic logistic distribution model, respectively.

**Figure 4:**
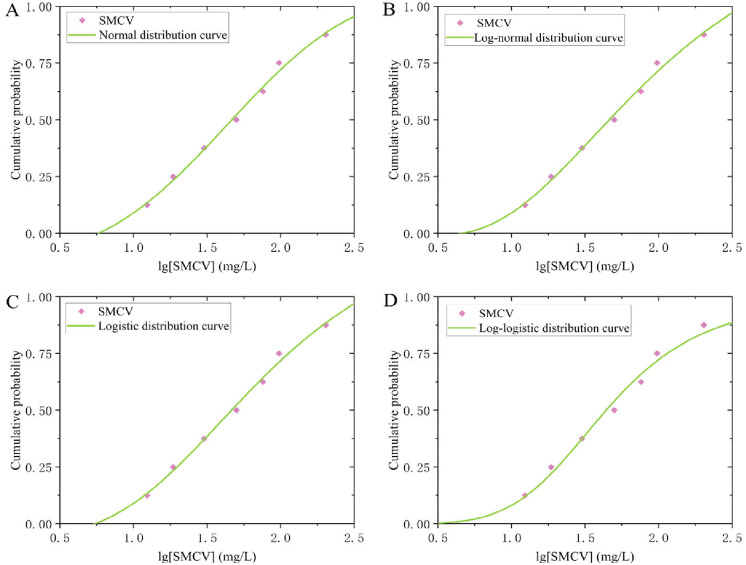
Fitting curves of four models to lg (SMCV) data. (**A**–**D**) represent the fitting curves using the normal distribution model, logarithmic normal distribution model, logistic distribution model, and logarithmic logistic distribution model, respectively.

Given the broad applicability of fitting models, the normal, log-normal, logistic, and log-logistic distributions were selected for analysis. Normality was assessed using the Shapiro–Wilk and Anderson–Darling tests, chosen for their reliability with the available sample sizes [37]. The tests were applied to 34 species mean acute values (SMAVs) and 7 species mean chronic values (SMCVs). The results indicated that the raw SMAV data were not normally distributed (*p* < 0.05), whereas the raw SMCV data met the normality assumption (*p* > 0.05). After applying a base-10 logarithmic transformation to both datasets, normality tests were repeated on lg (SMAV) and lg (SMCV). Both transformed datasets exhibited normality (*p* > 0.05), with reduced standard deviations (S.D.), lower Anderson–Darling values, skewness and kurtosis closer to 0, and W values closer to 1 compared to the untransformed data. Consequently, the log-transformed data were more suitable for subsequent parameter fitting. A comparative summary of these results is presented in Table 3. The lg (SMAV) and lg (SMCV) datasets were fitted using the National Ecological Environment Criteria Calculation Software—Species Sensitivity Distribution (EEC-SSD) method. Results indicated that the normal distribution model provided the best fit for both acute data (RMSE = 0.04671, *p* > 0.05) and chronic data (RMSE = 0.05820, *p* > 0.05). Although the log-normal and log-logistic distributions were identified as ecologically plausible and showed good fit based on the Akaike Information Criterion (AIC) and visual assessment, the HC_5_ values derived from these models remained higher than those obtained from the normal distribution [38]. Given that the study aims to propose a regulatory recommendation value, this outcome is not ideal. Therefore, the HC5 value generated by the normal distribution was selected for subsequent calculations. The detailed fitting results of the four models for acute and chronic data are shown in Figure 3 and Figure 4, and Table 4. The normal distribution model was used for lg (SMAV) data, with a corresponding HC_5_ value of 34.94 mg/L. As the number of species is greater than 15, the AF value is 2, and the calculated SWQC is 17.47 mg/L. The lg (SMCV) data were analyzed using a normal distribution model, with a corresponding HC_5_ value of 10.00 mg/L. Since the number of species is less than 15, the AF value is 3, and the calculated LWQC is 3.334 mg/L.

### 3.3. Ecological Risk Assessment

China was selected as a representative region for ecological risk assessment of fluoride (as F^−^). Surface water concentration data of fluoride were compiled from 21 provincial-level administrative regions and 32 major river and lake basins (including tributaries) across China, covering the period from 1986 to 2023. According to the current Chinese Surface Water Environmental Quality Standard (GB 3838-2002), among the 1445 data points collected, 1153 (approximately 79.79%) fell within the Class I–III limit (≤1.0 mg/L), while 163 (approximately 11.28%) fell within the Class IV–V limit (>1.0 mg/L and ≤1.5 mg/L). The collected data were preprocessed, and a preliminary decision was made on whether to use the mean or median for subsequent calculations. The standard deviation and coefficient of variation (CV) of the concentration data are summarized in Table 5.

Based on the calculated CV, only 20 out of the 53 concentration datasets exhibited a CV below 20%. This indicates high variability within the data, justifying the use of the median rather than the mean for subsequent calculations.

Among the compiled fluoride concentration data, the Xihe River Basin exhibited the highest median concentration (21.09 mg/L), while the Ertix River Basin showed the lowest (0.18 mg/L). At the provincial level, Anhui Province recorded the highest median fluoride content (1.14 mg/L), and Guangxi Zhuang Autonomous Region the lowest (0.00038 mg/L). The Shahe River Basin exhibited the widest fluoride concentration range (0.05–181.6 mg/L; a 3632-fold difference), whereas the Wujiang River Basin showed the narrowest range (0.2–0.21 mg/L; a 1.05-fold difference). At the provincial level, the concentration range was greatest in Fujian (0.12–12.5 mg/L; 104.17-fold) and smallest in Heilongjiang (0.333–0.37 mg/L; 1.11-fold).

Comparison with the SWQC and LWQC derived from the species sensitivity distribution method revealed that the median fluoride concentrations in Ebinur Lake, Chagan Lake, and the Shahe River exceeded the LWQC, suggesting a potential chronic toxic effect on aquatic organisms in these water bodies. In contrast, the median concentration in the Xihe River surpassed both the SWQC and LWQC, indicating a risk of both acute and chronic toxicity to its aquatic biota.

**Figure 5:**
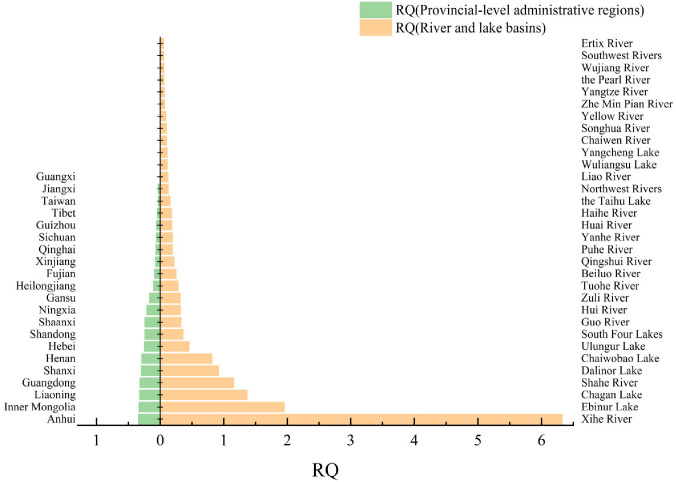
Comparison of RQ values among river basins and provincial-level administrative regions.

All concentration data were evaluated using the LWQC derived from the species sensitivity distribution method. The resulting RQ values and corresponding risk levels are presented in Table 6 and Figure 5. Among river/lake basins, the Xihe River Basin showed the highest RQ (6.326, high risk), while the Ertix River Basin had the lowest (0.05399, low risk). At the provincial level, Anhui Province exhibited the highest RQ (0.3419, moderate risk), and Guangxi Zhuang Autonomous Region the lowest (0.0001140, no risk). Four basins were classified as high risk: Xihe River (RQ = 6.326), Ebinur Lake (1.953), Chagan Lake (1.368), and Shahe River (1.158). The remaining basins were rated as moderate or low risk, and those without basin-specific data were categorized as no risk. At the provincial scale, no region was assessed as high risk. Most provinces fell into moderate or low risk categories, with Guangxi Zhuang Autonomous Region being the only one classified as no risk.

### 3.4. Comparison of Fluoride Water Quality Standards in Different Countries

Many countries in the world have limits on the concentration of fluoride in water bodies. Under the principle of phased development of chemical pollutant regulations, the United States Environmental Protection Agency (U.S. EPA) has included 65 pollutants, including inorganic fluorides, in the second/fifth phase of chemical pollutant regulations, and included them in Maximum Contaminant Level Goals (MCLG), Maximum Contaminant Levels (MCL), monitoring requirements and removal technologies [39]. Canada has set fluoride limits of 1.5 mg/L and 0.12 mg/L, respectively, in the “Guidelines for Canadian Drinking Water Quality” and the “Water Quality Guidelines for the Protection of Aquatic Life” [40,41]. However, there is not much research on the criteria of fluoride in freshwater aquatic organisms by domestic and foreign scholars. McPherson et al. collected chronic toxicity data of 16 species in 2014, and based on this, used the species sensitivity distribution method to derive a chronic baseline of 1.4 mg/L for fluoride. In their study, they pointed out that there is currently a lack of international criteria research on fluoride [42]. In the process of demonstrating the practicality of the empirical bioavailability model based on multiple linear regression, Parker et al. introduced three toxicity-modifying factors (TMFs) and obtained a preliminary final acute value (FAV) (16 species) range of 18.1 mg/L–56.3 mg/L. Then, using acute to chronic ratio (ACR), the range of final chronic value (FCV) was calculated to be 3.4 mg/L–10.4 mg/L; When TMFs is not introduced, FAV (29 species), FCV (19 species), and FCV derived through ACR are 30.6 mg/L, 4 mg/L, and 5.7 mg/L, respectively; The team pointed out in their research that there is a lack of uniformity in the species selection required for model construction, resulting in differences in the sensitivity of the same species to fluoride observed. According to research findings, some indicator values (such as AF and ACR) in the calculation methods used in guidelines of countries like the United States and Canada deviate from academic consensus, potentially leading to a lack of objectivity in fluoride limits [43,44,45]. Comparing the criteria values derived using the species sensitivity distribution method with domestic and international standards (see Table 7), it was found that the derived LWQC values were only lower than the criteria standards in the United States. This may be due to the fact that species are distributed all over the world in this criteria derivation study, and chronic toxicity values are relatively lacking. The resulting SMCV data only have 7 and do not cover the number of species to 3 phyla and 8 families, making it impossible to use methods that require the number of species covered by phyla and families when deriving the criteria.

## 4. Uncertainty Analysis

### 4.1. Toxicity Data Compilation and Water Quality Criteria Derivation

The limited availability of chronic toxicity data undermines the reliability of the derived water quality criteria. To ensure ecological representativeness, such criteria must be based on toxicity data that adequately cover the sensitivity of diverse species to pollutants. For example, U.S. guidelines require data spanning three phyla, eight families, and at least one aquatic plant, while Chinese regulations stipulate data from ten species across three trophic levels, including one cyprinid fish [47]. As this study focuses on ecological risk assessment in China, the chronic toxicity data collected here clearly do not meet the national regulatory requirements. Furthermore, regarding species selection, only invasive species were excluded from the analysis due to data scarcity. This limited exclusion may result in derived water quality criteria that do not fully address the protection needs of China’s native species.

In terms of pollutant sources, the available toxicity data are limited to three fluoride compounds: sodium fluoride (NaF), ammonium fluoride (NH_4_F), and aluminum fluoride (AlF_3_), with the majority originating from NaF. This pattern reflects a prevalent preference in experimental design, as researchers often select NaF for fluoride toxicity studies in aquatic organisms. This tendency may be explained by the relatively low toxicity of sodium ions to aquatic life, as demonstrated in the study by Camargo, which used NaCl controls to isolate fluoride-specific toxicity in trout species [48]. Furthermore, multiple water quality parameters—including pH, hardness, alkalinity, chloride concentration, and temperature—have been reported to modulate fluoride toxicity [49]. Since this study did not normalize toxicity data for variations in these parameters, we recommend that future efforts incorporate such adjustments to minimize confounding effects and enhance the reliability and comparability of toxicity data.

In terms of bioavailability, numerous studies have indicated that freshwater fish exhibit greater tolerance to fluoride toxicity in soft water than in hard water [50,51]. This phenomenon can be explained by two primary mechanisms based on current evidence. First, in aquatic environments, polyvalent cations such as Ca^2+^ and Mg^2+^ can react with fluoride ions to form insoluble precipitates, thereby reducing fluoride bioavailability. Second, after entering a fish’s body, fluoride ions tend to form stable complexes with calcium present in blood or bone tissues [52,53].

Regarding the selection of the SSD model, although the log-normal and log-logistic distributions are often considered the most statistically reliable, the HC_5_ values derived from these models remained comparatively high. Given the limited quality of the available toxicity data, opting for the normal distribution—which yields a more conservative HC_5_ estimate—presents a more prudent choice for protective risk assessment.

### 4.2. Ecological Risk Assessment

Several limitations should be acknowledged regarding the fluoride surface water concentration data compiled in this study. First, the temporal span of data in some regions is extensive, and the inclusion of historical concentrations from earlier periods may affect the representativeness of values used for ecological risk assessment. In regions with limited sample sizes, data are more vulnerable to temporal and spatial variability. Second, sample sizes in many areas are limited, which may not accurately reflect recent fluoride levels. Furthermore, the high standard deviation and coefficient of variation indicate considerable data dispersion and the presence of outliers across multiple regions. Under such conditions, the use of the median or percentiles as representative values for subsequent calculations is more appropriate than the mean.

Additionally, the RQ method has inherent constraints. Its outcome depends heavily on the toxicity benchmark adopted. In this study, LWQC was derived from only seven species, which may affect its stability and, consequently, the reliability of the RQ estimates. The MEC value also significantly influences RQ calculations, meaning that RQ values fluctuate with monitoring data and may not fully capture dynamic risk trends. Therefore, if the RQ method is applied for ecological risk assessment, then repeated follow-up monitoring of local concentrations is recommended to update risk evaluations effectively.

## 5. Conclusions

(1) Toxicity data for fluorides were collected from both domestic and international sources. The acute dataset included 34 species across 14 families and 4 phyla, while the chronic dataset comprised 7 species from 5 families and 3 phyla. Using the species sensitivity distribution (SSD) method, the derived short- and long-term water quality criteria (SWQC and LWQC) were 17.47 mg/L and 3.334 mg/L, respectively. Comparison with existing standards from various countries, regions, and organizations suggested that data scarcity was the primary factor limiting the objectivity of the SWQC and LWQC derived in this study.

(2) China was selected as the study area for ecological risk assessment using the risk quotient (RQ) method. The results indicated relatively high ecological risk in the Xihe River, Ebinur Lake, Chagan Lake, and Shahe River basins. Among provincial-level administrative regions, Guangxi Zhuang Autonomous Region was assessed as no risk, and no region was classified as high risk. Overall, risk levels across both basins and administrative regions were predominantly categorized as moderate or low.

## 6. Limitations and Future Perspectives

Based on the current research, several limitations exist in the freshwater water quality criteria and ecological risk assessment of fluoride. The most prominent issue is data scarcity. The quantity and quality of available data fundamentally determine the reliability of research outcomes. In recent years, studies on freshwater toxicity exposure experiments and surface water concentration monitoring of fluoride have been limited, which has significantly constrained data availability. This shortfall adversely affects the entire decision-making chain for fluoride management. Furthermore, many studies do not adequately adhere to region-specific criteria when selecting toxicity data, resulting in poor representation of local ecosystems and poor alignment between the spatiotemporal scales of concentration data and toxicity data. Even after excluding unsuitable data, the remaining dataset often remains too small to ensure objective and robust conclusions, highlighting the persistent deficiency in fluoride-related toxicity and environmental concentration data.

In terms of methodology, insufficient attention is often given to the statistical significance of key indicators. It is not uncommon for certain metrics to be adjusted through arbitrary settings to achieve apparent improvements or innovations, without proper comparison to established methods or discussion of their applicability.

To address these issues, the authors recommend first strengthening data infrastructure. This includes conducting continuous experiments to expand data volume and enhancing the development of localized species databases to improve data sharing and support subsequent research. Additionally, different machine learning models (e.g., random forest regression, RFR) could be employed to predict temporal trends in fluoride concentrations and their correlation with various environmental parameters, or to analyze the role of key indicators within statistical models, thereby ensuring efficient data processing and high-quality analytical outcomes.

## Figures and Tables

**Table 1:** Acute toxicity data of fluoride to freshwater organisms.

Phylum	Family	Species Common Name	Species Scientific Name	Effect	Endpoint	SMAV (mg/L)
Chordata	Acipenseridae	*Siberian sturgeon*	*Acipenser baeri*	Mortality	LC_50_	392.4
		*Chinese sturgeon*	*Acipenser sinensis*	Mortality	LC_50_	131.74
	Cyprinidae	*Catla*	*Catla catla*	Mortality	LC_50_	53.37
		*Mrigal*	*Cirrhinus mrigala*	Mortality	LC_50_	505.56
		*Fathead minnow*	*Pimephales promelas*	Mortality	LC_50_	310.24
		*Stigma barb*	*Puntius sophore*	Mortality	LC_50_	129.48
	Salmonidae	*Silver salmon*	*Oncorhynchus kisutch*	Mortality	MATC	500
		*Rainbow trout*	*Oncorhynchus mykiss*	Mortality	LC_50_	134.74
		*Brown trout*	*Salmo trutta*	Mortality	LC_50_	83.89
	Bufonidae	*Asiatic toad*	*Bufo gargarizans*	Mortality	LC_50_	610.44
	Channidae	*Spotted snakehead*	*Channa punctata*	Mortality	LC_50_	300
	Gasterosteidae	*Threespine stickletback*	*Gasterosteus aculeatus*	Mortality	LC_50_	390.25
	Centrarchidae	*Bluegill*	*Lepomis macrochirus*	Mortality	LC_50_	663.25
	Poeciliidae	*Guppy*	*Poecilia reticulate*	Mortality	LC_50_	248.65
Arthropoda	Hydropsychidae		*Hydropsyche exocellata*	Mortality	LC_50_	46.46
			*Hydropsyche occidentalis*	Mortality	LC_50_	40.53
			*Hydropsyche bronta*	Mortality	LC_50_	20.43
			*Hydropsyche pellucidula*	Mortality	LC_50_	63.4
			*Hydropsyche lobata*	Mortality	LC_50_	61.39
			*Cheumatopsyche pettiti*	Mortality	LC_50_	49.84
			*Hydropsyche bulbifera*	Mortality	LC_50_	45.39
	Stenopsychidae		*Chimarra marginata*	Mortality	LC_50_	75.44
	Daphnidae		*Ceriodaphnia dubia*	Mortality	EC_50_	157.9
			*Ceriodaphnia pulchella*	Mortality	EC_50_	83.2
			*Daphnia carinata*	Mortality	EC_50_	353.6
			*Simocephalus vetulus*	Mortality	EC_50_	210.5
		*Large water flea*	*Daphnia magna*	Mortality	EC_50_	294.97
					LC_50_	
				Survival	EC_50_	
				Growth	EC_50_	
Mollusca	Unionidae		*Actinonaias pectorosa*	Mortality	LC_50_	237.07
		*Appalachian elktoe*	*Alasmidonta raveneliana*	Mortality	LC_50_	303
		*Wavyrayed lampmussel*	*Lampsilis fasciola*	Mortality	LC_50_	133
		*Paper pondshell*	*Utterbackia imbecillis*	Mortality	LC_50_	234
	Viviparidae		*Bellamya aeruginosa*	Mortality	LC_50_	132.74
			*Cipangopaludina cathayensis*	Mortality	LC_50_	583.8
Chlorophyta	Chlorellales	*Chlorella*	*Chlorella vulgaris*	Mortality	LC_50_	120.86
				Growth	LC_50_	

**Table 2:** Chronic toxicity data of fluoride to freshwater organisms.

Phylum	Family	Species Common Name	Species Scientific Name	Effect	Endpoint	SMCV (mg/L)
Chordata	Salmonidae	*Rainbow trout*	*Oncorhynchus mykiss*	Mortality	MATC	75.78
					LC_50_	
		*Brown trout*	*Salmo trutta*	Mortality	LC_50_	97.5
	Acipenseridae	*Siberian sturgeon*	*Acipenser baeri*	Growth	LOEC	12.37
					NOEC	
	Cyprinidae	*Zebra danio*	*Danio rerio*	Growth	LOEC	18.6
Arthropoda	Daphniidae		*Daphnia carinata*	Reproduction	LOEC	50
		*Large water flea*	*Daphnia magna*	Mortality	NOEC	30.15
				Growth	NOEC	
				Reproduction	LOEC	
					NOEC	
Chlorophyta	Chlorellales	*Chlorella*	*Chlorella vulgaris*	Mortality	LC_50_	160.82
				Growth	LC_50_	
				Population	NOEC	

**Table 3:** Comparison of normality test results for normal distribution, log-normal distribution, logistic distribution, and log-logistic distribution.

Item	*n*	Mean (mg/L)	S.D.	Skewness	Kurtosis	Anderson–Darling		Shapiro–Wilk	
						AD	*p* Value	W	*p* Value
SMAV	34	226.3	182.1	0.959	−0.039	1.352	0.001	0.882	0.002
lg (SMAV)	34	2.198	0.398	−0.254	−0.849	0.423	0.302	0.962	0.277
SMCV	7	69.60	66.29	1.598	2.7	0.497	0.137	0.842	0.103
lg (SMCV)	7	1.674	0.425	0.074	−0.887	0.133	0.958	0.983	0.971

**Table 4:** Fitting results of four models for acute and chronic data.

	Selected Model	HC_5_ (mg/L)	RMSE	*p* (Anderson–Darling Test)
lg (SMAV)	Normal distribution	34.94	0.04671	>0.05
	Log-normal distribution	37.94	0.05221	>0.05
	Logistic distribution	32.92	0.04908	>0.05
	Log-logistic distribution	38.14	0.05113	>0.05
lg (SMCV)	Normal distribution	10.00	0.05820	>0.05
	Log-normal distribution	11.62	0.06422	>0.05
	Logistic distribution	10.01	0.06875	>0.05
	Log-logistic distribution	11.99	0.07106	>0.05

**Table 5:** The fluoride concentration (expressed as F^−^) in surface water in China.

Provincial-Level Administrative Region/River and Lake Basin	Time	*n*	Concentration Range (mg/L)	Mean Value (mg/L)	Median Value (mg/L)	S.D.	CV (%)
Yangtze River	2004–2020	31	0.12–0.29	0.2224	0.22	0.05282	23.75
Yellow River	1986–2021.7	65	0.06–1.79	0.4419	0.3	0.3359	76.01
Zhe Min Pian River	2020	1	0.239	0.239	0.239	0	0
Northwest Rivers	2020	1	0.428	0.428	0.428	0	0
Southwest Rivers	2020	1	0.19	0.19	0.19	0	0
Songhua River	2020	1	0.325	0.325	0.325	0	0
the Pearl River	2020	1	0.205	0.205	0.205	0	0
Wujiang River	1989–1999	2	0.2–0.21	0.205	0.205	0.005	2.439
Liao River	2020	1	0.41	0.41	0.41	0	0
Shahe River	1986–2004	20	0.05–181.6	25.49	3.86	47.95	188.1
Puhe River	2018–2022	5	0.25–0.72	0.58	0.64	0.1726	29.76
Qingshui River	2013–2022	102	0.29–1.84	0.8127	0.725	0.3541	43.57
Huai River	2020	1	0.61	0.61	0.61	0	0
Haihe River	2020	18	0.5–3.72	0.9034	0.6	0.8729	96.62
Guo river	2009.1–2020.9	23	0.882–1.51	1.130	1.09	0.1522	13.47
Tuohe River	2009.1–2023	7	0.84–1.171	1.011	0.943	0.1166	11.53
Hui River	2009.1–2020.9	3	1.006–1.215	1.094	1.06	0.08858	8.097
Chaiwen River	2007.1–2016.9	376	0.16–0.65	0.3260	0.33	0.07284	22.34
Beiluo River		2	0.66–1	0.83	0.83	0.17	20.48
Yanhe River		2	0.64	0.64	0.64	0	0
Xihe River	1986–2004	8	0.57–42.72	19.18	21.09	15.87	82.74
Ertix River	2021.4–2021.10	1	0.18	0.18	0.18	0	0
Zuli River		1	1.048	1.048	1.048	0	0
Wuliangsu Lake	2021.7	16	0.19–1.9	0.5039	0.375	0.4439	88.09
the Taihu Lake	2010.5.22–2010.6.10	1	0.52	0.52	0.52	0	0
Yangcheng Lake	2018.11.5–2018.11.7	55	0.15–0.5	0.3551	0.37	0.09326	26.26
Chagan Lake	2008	1	4.56	4.56	4.56	0	0
South Four Lakes	2022.2–2022.3	1	1.2	1.2	1.2	0	0
Dalinor Lake	2016–2021	6	1.62–4.24	2.892	3.06	0.9466	32.73
Ebinur Lake	2005–2018	13	2.31–16.9	7.208	6.51	4.516	62.65
Chaiwobao Lake	2005–2009	15	0.24–3.78	2.607	2.71	0.7727	29.64
Ulungur Lake	2021.4–2021.10	2	0.41–2.62	1.515	1.515	1.105	72.94
Anhui	2006–2020.9	38	0.77–7.75	1.703	1.14	1.541	90.49
Fujian	1997–2008	178	0.12–12.5	0.6450	0.3	1.480	229.5
Gansu	2011–2015	34	0.284–1.2	0.5584	0.55	0.2585	46.29
Guangdong	2010–2024	8	0.4–1.5	1.020	1.064	0.4850	47.55
Guangxi	2005	12	0.00024–0.00308	0.000642	0.00038	0.0007610	118.5
Guizhou	2013.7	1	0.19	0.19	0.19	0	0
Hebei	2013–2021	16	0.24–1.488	0.8222	0.825	0.4288	52.15
Heilongjiang		4	0.333–0.37	0.347	0.3425	0.01437	4.141
Henan	2009–2022.8	52	0.28–5.55	1.436	0.96	1.223	85.17
Jiangxi		15	0.068–0.231	0.1118	0.102	0.04286	38.34
Liaoning	2018–2023.2	8	0.453–1.17	0.9794	1.077	0.2424	24.75
Inner Mongolia	2008–2021	98	0.38–7.13	1.325	1.11	0.8398	63.38
Ningxia	2013.4.1–2013.4.26	4	0.4–1.4	0.8	0.7	0.3937	49.21
Qinghai	2005–2019	7	0–0.3	0.2186	0.23	0.09891	45.25
Shaanxi	2006–2018	79	0.15–5.18	1.028	0.8	0.8547	83.14
Shandong	2005–2021.8	66	0.25–1.49	0.7958	0.8015	0.2665	33.49
Shanxi	2013.4.1–2020	5	0.4–1.7	1.058	0.99	0.5202	49.17
Sichuan	2005–2012	4	0.17–0.22	0.78	0.195	0.025	3.205
Taiwan	2007	4	0.09–0.15	0.1125	0.105	0.02278	20.25
Xinjiang	2019.7–2019.8	7	0.144–1.83	0.4569	0.241	0.5627	123.2
Tibet	2013.4.27–2013.8.23	22	0.02–0.58	0.14	0.135	0.1293	92.36

**Table 6:** Ecological risk assessment results of surface water in various river and lake basins and provincial-level administrative regions in China.

River and Lake Basin	RQ	Assessment Result	Provincial-Level Administrative Region	RQ	Assessment Result
Xihe River	6.326	High risk	Anhui	0.3419	Moderate risk
Ebinur Lake	1.953	High risk	Inner Mongolia	0.3329	Moderate risk
Chagan Lake	1.368	High risk	Liaoning	0.3230	Moderate risk
Shahe River	1.158	High risk	Guangdong	0.3191	Moderate risk
Dalinor Lake	0.9178	Moderate risk	Shanxi	0.2969	Moderate risk
Chaiwobao Lake	0.8128	Moderate risk	Henan	0.2879	Moderate risk
Ulungur Lake	0.4544	Moderate risk	Hebei	0.2475	Moderate risk
South Four Lakes	0.3599	Moderate risk	Shandong	0.2404	Moderate risk
Guo river	0.3269	Moderate risk	Shaanxi	0.2400	Moderate risk
Hui River	0.3179	Moderate risk	Ningxia	0.2100	Moderate risk
Zuli River	0.3143	Moderate risk	Gansu	0.1650	Moderate risk
Tuohe River	0.2828	Moderate risk	Heilongjiang	0.1027	Moderate risk
Beiluo River	0.2490	Moderate risk	Fujian	0.08998	Low risk
Qingshui River	0.2175	Moderate risk	Xinjiang	0.07229	Low risk
Puhe River	0.1920	Moderate risk	Qinghai	0.06899	Low risk
Yanhe River	0.1920	Moderate risk	Sichuan	0.05849	Low risk
Huai River	0.1830	Moderate risk	Guizhou	0.05699	Low risk
Haihe River	0.1800	Moderate risk	Tibet	0.04049	Low risk
the Taihu Lake	0.1560	Moderate risk	Taiwan	0.03149	Low risk
Northwest Rivers	0.1284	Moderate risk	Jiangxi	0.03059	Low risk
Liao River	0.1230	Moderate risk	Guangxi	0.0001140	No risk
Wuliangsu Lake	0.1125	Moderate risk			
Yangcheng Lake	0.1110	Moderate risk			
Chaiwen River	0.09898	Low risk			
Songhua River	0.09748	Low risk			
Yellow River	0.08998	Low risk			
Zhe Min Pian River	0.07169	Low risk			
Yangtze River	0.06599	Low risk			
the Pearl River	0.06149	Low risk			
Wujiang River	0.06149	Low risk			
Southwest Rivers	0.05699	Low risk			
Ertix River	0.05399	Low risk			

**Table 7:** The comparison results between the derived results of this study and the fluoride water quality standards of various countries/regions/organizations.

	Countries/Regions/Organizations	Water Source Type	Classification	F^−^ (mg/L)
This study		Surface water		17.47 (SWQC)
				3.334 (LWQC)
Surface Water Environment Quality Standard (GB 3838-2002) *	China	Groundwater	I	≤1.0
			II	≤1.0
			III	≤1.0
			IV	≤1.5
			IV	≤1.5
Groundwater Quality Standard (GB/T 14848-2017) *	China	Groundwater	I	≤1.0
			II	≤1.0
			III	≤1.0
			IV	≤2.0
			IV	>2.0
Standard for Drinking Water Quality (GB 5749-2022) *	China	Drinking water		1.0
Guidelines for drinking water quality: fourth edition incorporating the first and second addenda *	WHO	Drinking water		≤1.5
National Primary Drinking Water Regulations (NPDWR) *	USA	Drinking water		4.0 (MCL ①)
Australian Drinking Water Guidelines Version 4.0 *	Australia	Drinking water		≤1.5
Water Services (Drinking Water Standards for New Zealand) Regulations 2022 *	New Zealand	Drinking water		≤1.5
(EU) 2020/2184 * [46]	EU	Drinking water		≤1.5
Environmental Quality Standards (EQS) *	Japan	Groundwater		≤0.8
Revision of Drinking Water Quality Standards in Japan *	Japan	Drinking water		≤0.8
GROUNDWATER STANDARDS	Malaysia	Groundwater		1.5
Surface and Groundwater Quality Control Regulation-2011	Nigeria	Groundwater		0.6–1.5 (HDL ②)
				1.5 (MPL ③)
Water Quality Standards for drinking water in Korea *	Korea	Drinking water		≤1.5
Guidelines for Canadian Drinking Water Quality *	Canada	Drinking water		1.5 (MAC ④)
Water Quality Guidelines for the Protection of Aquatic Life *	Canada			0.12
2024 Update of the national groundwater quality indicator *	New Zealand	Groundwater		0.01–0.41

The symbol ‘*’ indicates that this criteria standard is in force. ① MCL: maximum contaminant levels; ② HDL: highest disirable level; ③ MPL: maximum permissible level; ④ MAC: maximum acceptable concentrations.

## Data Availability

The original contributions presented in this study are included in the article.

## Abbreviations

The following abbreviations are used in this manuscript:

ACR	Acute to Chronic Ratio
AF	Assessment Factor
AIC	Akaike Information Criterion
AlF_3_	Aluminum Fluoride
AVE	Acute Value for the same Effect
CV	Coefficient of Variation
CVE	Chronic Value for the same Effect
ECx	x% Effect Concentration
EEC-SSD	National Ecological Environment Criteria Calculation Software-Species Sensitivity Distribution Method
FAV	Final Acute Value
FCV	Final Chronic Value
HC_x_	Hazardous Concentration for x% of species
HC_5_	Hazardous Concentration for 5% of species
HDL	Highest Disirable Level
LCx	x% Lethal Concentration
LOEC	Lowest Observed Effect Concentration
LWQC	Long-term Water Quality Criteria for aquatic organisms
MAC	Maximum Acceptable Concentrations
MATC	Maximum Acceptable Toxicant Concentration
MCL	Maximum Contaminant Levels
MCLG	Maximum Contaminant Level Goals
MEC	Measured Environmental Concentration
MPL	Maximum Permissible Level
NaF	Sodium Fluoride
NH_4_F	Ammonium Fluoride
NHANES	National Health And Nutrition Examination Survey
NOEC	No Observed Effect Concentration
PNEC	Predicted No-Effect Concentration
RFR	Random Forest Regression
RMSE	Root Mean Square Error
RQ	Risk Quotient
S.D.	Standard Deviations
SMAV	Species Mean Acute Value
SMCV	Species Mean Chronic Value
SSD	Species Sensitivity Distribution
SWQC	Short-term Water Quality Criteria for aquatic organisms
TMFs	Toxicity-Modifying Factors
U.S. EPA	United States Environmental Protection Agency
WQC	Water Quality Criteria for aquatic organisms
